# Intraoperative validation of Left internal mammary artery Graft by Fluorescence Imaging Technique

**DOI:** 10.1186/1749-8090-10-S1-A1

**Published:** 2015-12-16

**Authors:** MG Kibria, AM Asif Rahim, MAH Pervez, T Ahmed, MSH Talukder, A Zaman, QM Hoque

**Affiliations:** 1 Department of Cardiovascular Surgery, National Institute of Cardiovascular Disease, Dhaka, Bangladesh

## Background/Introduction

In Surgical revascularization by CABG surgery Left internal mammary artery (LIMA) to Lt.ant.descending (LAD) is considered gold standard conduit of choice for myocardial revascularization. Although conventional angiography is gold standard for assessing graft patency but rarely available in operating room in Bangladesh. So intraoperative florescence imaging (Novadaq Technologies SPY TM System IFI) is an angiography like imaging using fluorescence indocyanine green excited laser light, (ICG) this device permits validation of graft patency intraoperatively.

## Aims/Objectives

To ensure patency after completion of graft and do revisions if necessary peroperatively when chest is open is mandatory for implementation of one of the techniques for intraoperative graft validation in Bangladesh. IFI is the safe, cost-effective, less time consuming and has no radiation risk. So the aim was to evaluate the intraoperative fluorescence imaging (IFI) system in the real-time assessment of graft patency during CABG to ensure the highest quality of the surgery and evaluate feasibility of the IFI to visualize flow in the native and grafted vessels in CABG specifically LIMA to LAD.

## Method

It was a descriptive cross sectional study at NICVD between July 2013 to June 2014 NICVD on Spy TM System (IFI) in CABG surgery focused on 36 patients' LIMA to LAD grafts.

## Results

Of the total 36 patient's mean age 54 ± 8.38 years, body mass index 25.87 ± 2.39. The quality of LIMA to LAD graft were assessed by using. Quality of anastomosis were assessed intraoperatively to validate graft. peroperative assessment of LIMA to LAD by IFI system revealed 32 (88.8%) patent anastomosis, Narrowing of anastomosis 2 (5.55%) stenosis of the anastomosis in 2(5.55%).

## Discussion/Conclusion

Using IFI system is a best way to validate patency of LIMA graft intraoperatively. Since it does not have any adverse effect may become the gold standard for surgical management in the near future.

